# Chromothripsis—Explosion in Genetic Science

**DOI:** 10.3390/cells10051102

**Published:** 2021-05-04

**Authors:** Mariia Shorokhova, Nikolay Nikolsky, Tatiana Grinchuk

**Affiliations:** Department of Intracellular Signaling and Transport, Institute of Cytology, Russian Academy of Sciences, Tikhoretskay Ave 4, 194064 St. Petersburg, Russia; cellbio@incras.ru (N.N.); grintat@bk.ru (T.G.)

**Keywords:** chromothripsis, chromosomal instability, micronuclei, cancer, transformation

## Abstract

Chromothripsis has been defined as complex patterns of alternating genes copy number changes (normal, gain or loss) along the length of a chromosome or chromosome segment (International System for Human Cytogenomic Nomenclature 2020). The phenomenon of chromothripsis was discovered in 2011 and changed the concept of genome variability, mechanisms of oncogenic transformation, and hereditary diseases. This review describes the phenomenon of chromothripsis, its prevalence in genomes, the mechanisms underlying this phenomenon, and methods of its detection. Due to the fact that most often the phenomenon of chromothripsis occurs in cancer cells, in this review, we will separately discuss the issue of the contribution of chromothripsis to the process of oncogenesis.

## 1. Introduction

The genome (in particular the karyotype) is a complex system, the well-functioning of which ensures the correct operation of the whole body. Aberrations in the karyotype, both structural and numerical, can lead to malfunctioning of the genome and cause tumor cell transformation. Tumor cell transformation is usually accompanied by numerical (monosomies, trisomies, tetrasomies, etc.) and structural chromosomal alterations, including: chromatid breaks, ring chromosomes, additional material of unknown origin, derivatives chromosomes, balanced translocations, deletions, inversions and non-condensed whole chromosomes, or chromosomes with impaired heterochromatin condensation, among others [1,2,3,4,5,6]. In the cancer cell genomes detected, the multiple chromosomal changes are the result of a stepwise process in which mutations accumulate over time [7].

Relatively recently, another form of karyotypic variation was discovered, associated with fragmentation (pulverization) of chromosomes [8].

Before considering in detail the phenomenon of chromothripsis, we decided to focus on its “brothers”, which have similar features to it. However, from a historical point of view, chromothripsis was discovered first, after which other types of complex chromosomal rearrangements were described.

## 2. Types of Multiple Complex Chromosomal Rearrangements

Before considering in detail the phenomenon of chromothripsis, it is necessary to pay attention to multiple chromosomal rearrangements. Holland and Cleveland proposed the term “*chromoanagenesis*” for all catastrophic events producing complex chromosomal rearrangements [9,10]. Chromoanagenesis is the event in which a large number of complex rearrangements occur in one or more chromosomal loci during one catastrophic event. Chromothripsis is a subset of chromoanagenesis.

**Figure 1:**
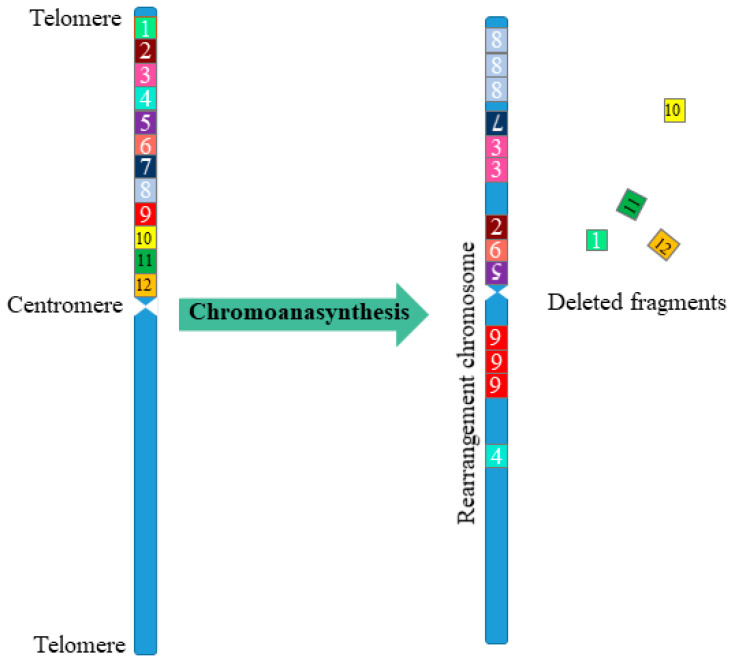
Scheme of multiple complex chromosomal rearrangements during chromoanasynthesis. In chromoanasynthesis, as a result of absent DNA replication, a part of the chromosome (fragments 1–12) undergoes complex rearrangements. In the rearranged chromosome, in addition to changing the sequence of the chromosome region (fragments 7, 2, 6, 5, 4), amplified regions are observed (fragments 8, 3, 9), and some of the fragments are removed (fragments 1, 10, 11, 12). The main difference between chromoanasynthesis and chromothripsis as well as chromoplexy is the presence of both losses and gains (duplications/triplications) region on the rearranged chromosome.

Alongside chromothripsis, an event has been identified, called *chromoanasynthesis*. The chromoanasynthesis based on a disorder of the replication process—a sequential stop of replication forks or a violation of replication mechanisms mediated by microhomology. During chromoanasynthesis, the lagging strand of the defective fork is decoupled, and a number of microhomologically dependent patterns and switching events occur with other replication forks. This process leads to the formation of complex genomic rearrangements, which usually involve duplications and triplications [11]. The key difference between chromoanasynthesis and chromothripsis lives in the presence of gene copies (two or three copies) in addition to deletions and normally copied regions of the chromosome (Figure 1).

**Figure 2:**
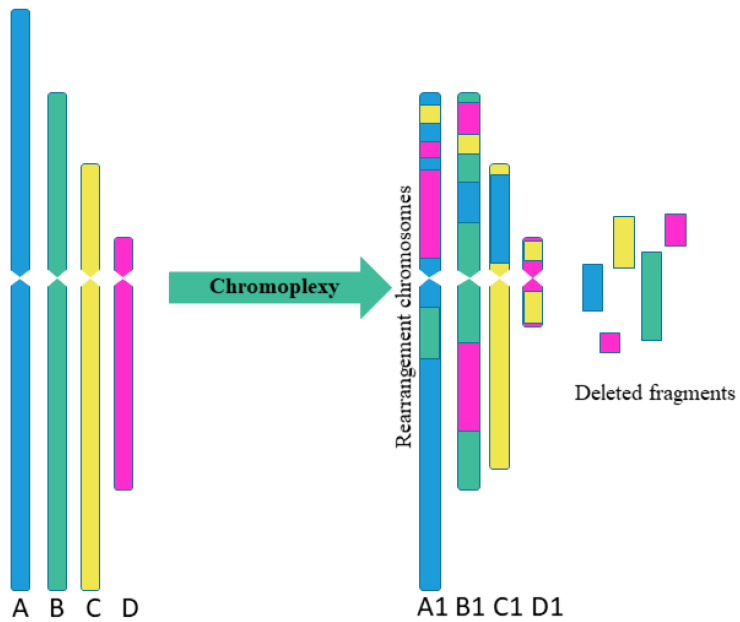
Scheme of multiple complex chromosomal rearrangements in chromoplexy. Chromoplexy involves linked translocations of multiple chromosomes. As a result of chromoplexy from chromosome A was formed rearranged chromosome A1, including fragments of chromosomes B, C, D (2 fragments); from chromosome B was formed rearranged chromosome B1, including fragments of chromosomes A, C, D (2 fragments); from chromosome C was formed rearranged chromosome C1, including fragment of chromosome A; from chromosome D was formed rearranged chromosome D1, including 2 fragments of chromosome C. Some fragments were delated.

Sequencing of prostate cancer led to the discovery of *chromoplexy*, another special type of complex chromosome rearrangement (Figure 2). The chromoplexy is characterized by the participation of several chromosomes in rearrangements [12]. The “chromoplexy”, derived from the Greek word “pleco”, which means “weave” or “braid”. The chromoplexy is defined as an accumulation of translocations involving several chromosomes (up to now, a maximum of 6 chromosomes have been detected simultaneously). The resulting chromosome rearrangements show little or no change in copy number, unlike chromothripsis, which is usually limited to one or two chromosomes and shows fluctuations between copy number. Chromosomes participating in chromoplexy often show fewer rearrangements than observed with chromothripsis. Another difference between chromothripsis and chromoplexy is the break–join point patterns. In chromoplexy, rearrangements are often found in the form of closed chains with almost exact contacts and almost without deletions [12,13]. As a result, the typical variation in the copy number observed with chromosomes after chromothripsis was not found for chromoplexy [14].

In this regard, when researchers detect multiple chromosomal changes, it is important to understand which type of rearrangements the researcher faces as the pattern of changes, mechanisms, and consequences of events differ. More likely, with the accumulation of knowledge about multiple chromosome rearrangements, their new types will be identified.

In later chapters, we will take thorough consideration of the phenomenon of chromothripsis.

## 3. Chromothripsis

**Figure 3:**
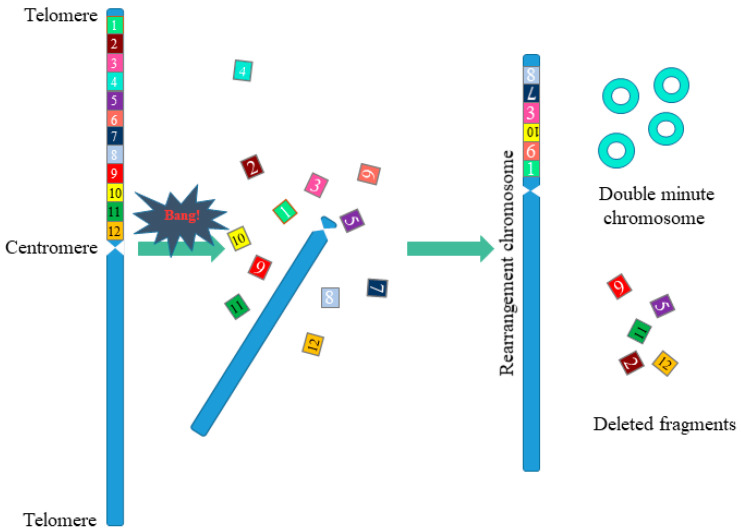
Scheme of chromosomal rearrangements in chromothripsis. During chromothripsis, the chromosome or the chromosome arm was destroyed, followed by incomplete and accidental restoration of fragments (1–12). When the chromosome is restored, the orientation of the fragments can change (fragments 7, 10), some of the fragments are removed (fragments 2, 5, 9, 11, 12), and some form extrachromosomal double minutes (fragments 4). This results in a rearranged chromosome or chromosome fragments.

Chromothripsis is a complex chromosomal rearrangement and is characterized by up to thousands of cluster chromosomal rearrangements that occur simultaneously and are localized in limited regions of the genome in one or several chromosomes. Thripsis in translation from Greek means destruction into small parts. Stephens with colleagues first described chromothripsis in 2011 when massive genome rearrangements were detected in patients with lymphocytic leukemia [8]. In this work, the authors were faced with an unusual case in which one of the patients had 42 genomic rearrangements localized in the long arm of chromosome 4, associated with chromosomal breaks at several points with the subsequent random assembly of fragments (Figure 3). Such a large-scale genomic change within one chromosomal arm did not fit into the classical mutational theory of oncogenesis.

The discovery of chromothripsis made it possible to establish the existence in nature of an “explosive” mechanism of rapid destabilization of the cellular genome. Initially, there has been much preliminary debate about the idea that a subset of the cancer genomes may not be related to the gradual evolution of the genome [15,16]. It is now generally accepted that chromothripsis is a widespread mutational phenomenon. A number of criteria were formulated to identify the rearrangements that occurred precisely as a result of chromothripsis [17]:

1. The genomes of cells with chromothripsis are characterized by clustered DNA breaks. Such clustered regions have multiple DNA breaks in close proximity to each other. These clustered regions are surrounded by large regions of DNA not affected by rearrangements.
2. As a result of chromothripsis, part of the chromosome fragments can be deleted.
3. Chromothripsis usually occurs on one parental copy (haplotype) of chromosomes.
4. Fragments of chromosomes obtained as a result of chromothripsis are combined in random order and have a random orientation.
5. After chromothripsis in a new rearranged chromosome, each fragment is either retained or lost.

As suggested by the authors themselves [17], these criteria are not exhaustive, but rather are intended to develop guidelines for the identification of chromothripsis and are subject to discussion. As this research area develops, new ideas are likely to emerge that will require reevaluation and adjustment of the chromothripsis characteristics. Since 2013, the International System for Human Cytogenetic Nomenclature has defined chromothripsis as complex patterns of alternating changes in the number of gene copies (normal, increased, or with loss) along a chromosome or chromosomal segment [18].

Information about the nature of chromothripsis is expanding every year. However, knowledge about this phenomenon is still far from complete, and the mechanisms of chromothripsis are not yet fully clear. In this review, we describe the prevalence of chromothripsis in nature, discuss its occurrence mechanisms, dwell on its detection methods, and discuss the chromothripsis role in the development of oncogenesis.

## 4. Mechanisms of Chromothripsis

**Figure 4:**
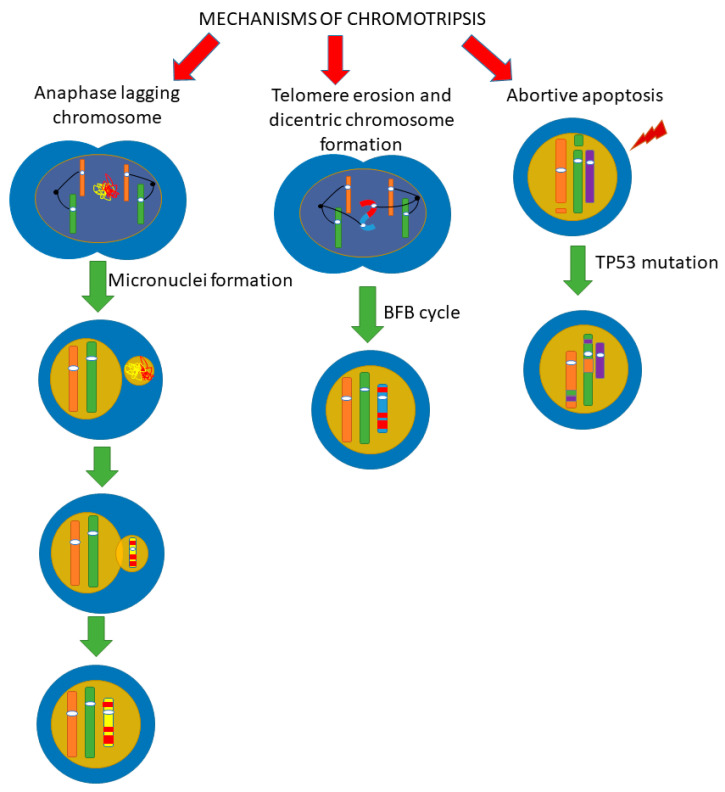
Scheme of possible pathways for chromothripsis. There are several pathways by which chromothripsis can be generated, involving abortive apoptosis, telomere erosion, and end-to-end fusion. The issue of implementing one mechanism or several at the same time is still under discussion. We believe that it is quite possible to implement several paths at once. (BFB cycle, breakage-fusion-bridge cycle).

Since the initial stage of chromothripsis is multiple DNA breaks, it has been firstly suggested that the cause of chromothripsis is the influence of severe exogenous stress factors that cause multiple double-stranded DNA breaks [19,20]. However, DNA damages caused by such triggers impact the entire genome, not just one chromosome or chromosomal region, as is observed in chromothripsis. The question about the “trigger” that causes chromothripsis is still open, and the mechanisms of the chromosomes fragmentation process with their subsequent assembly are currently being actively discussed. In recent years, several alternative mechanisms of chromothripsis have been proposed (Figure 4), which, however, are not mutually exclusive.

### 4.1. Fragmentation of Chromosomes in Micronuclei

Fragmentation of chromosomes in micronuclei is considered as one of the possible pathways for chromothripsis occurrence (Figure 4). Micronuclei are formed as a result of the fact that chromosomes or chromosome fragments lagging behind in mitosis are encapsulated by the nuclear envelope outside the main nucleus [21]. In this case, micronuclei are small round extra-nuclear structures consisting of DNA surrounded by a bilipid layer [22]. Both the whole segregated chromosome (the so-called lagging chromosomes) and its part can enter the micronuclei [23].

It has been demonstrated that a number of processes taking place in the main nucleus are absent or lagging behind in micronuclei, including DNA transcription and replication [24,25,26]. This can lead to double-stranded DNA breaks due to arrested or delayed replication forks [27]. In addition, DNA replication in the micronucleus is asynchronous with respect to the main nucleus [24,25]. In response to cytoplasmic mitotic signals, the micronucleus DNA can prematurely compact, resulting in multiple breaks in micronucleus DNA [24]. The further destiny of micronuclear DNA can be different. In the process of subsequent cell division, the micronucleus can be destroyed in the cytoplasm, which will result in the loss of micronuclear DNA. On the other hand, during subsequent cell division, the micronucleus and its DNA can be re-incorporated into the primary nucleus of one of the daughter cells.

There is strong experimental evidence linking chromothripsis to lagging chromosomes and micronucleus encapsulation [28]. Zhang and colleagues used a combination of live-cell imaging to track them after micronucleus induction using nocodazole-mediated microtubule depolymerization [29] followed by sequencing of individual daughter cells [28]. Micronuclear DNA could be identified in daughter cells due to the fact that it was not sufficiently replicated. Genomic analysis of the cells revealed de novo chromothriptic rearrangements on chromosomes that had apparently been previously encapsulated in the micronucleus. [28]. It was recently discovered that the cause of multiple DNA damage in micronuclei is mechanistic. The driving force behind multiple breaks is the dynamics of premature condensation of chromosomes in cells with asynchronous micronuclei [30].

### 4.2. Telomere Erosion and Dicentric Chromosome Formation

Another possible chromothripsis mechanism is associated with breakage–fusion–bridge (BFB) cycles (Figure 4) [8,31]. Chromothriptic breakpoints are often located within the telomere regions of the chromosome, indicating a possible role for telomere damage in the clustering of breakpoints [32]. Human telomeres consist of n-tandem repeats TTAGGG, limited by shelterin proteins. In somatic cells, in the process of normal division, the centromeres are shortened. This shortening occurs in every cell cycle. As a result, after a certain number of cycles, telomeres reach a critical length, and cell senescence is activated in the cell. However, if the *TP53* and *RB* genes are lost, cell cycle arrest does not occur in response to critical telomere shortening. As a result, these cells continue to multiply. The telomeres of these cells continue to shorten, eventually leading to dysfunctional and unprotected telomeres. When the protective functions of telomeres are lost, DNA repair mechanisms can initiate the fusion of telomeres and the formation of dicentric chromosomes. If a dicentric chromosome is formed in the cells, then later, during mitosis, chromatin bridges are formed, which subsequently rupture [33]. Thus, such cells enter BFB cycles. During anaphase, the centromeres of the dicentric chromosome can stretch in different directions of the dividing cell. The two ends of one dicentric chromosome find themselves between two daughter cells, due to which segregation cannot be completed and a chromatin bridge arises. Upon induction of envelope formation, the chromatin bridge is destroyed by the cytoplasmic 3’-exonuclease TREX1 [34,35]. This can lead to multiple losses and inversions of chromosomal segments and also to the formation of double minutes. The BFB cycle can occur simultaneously with the amplification of chromosome fragments [36]. In connection with this phenomenon, it was suggested that the BFB cycles associated with telomere shortening may lead to chromothripsis [8,31]. On the other hand, these cycles can be part of the formation of a new type of chromosome and be a consequence, not a cause, of chromothripsis [37], hence the relationship between BFB cycles and chromothripsis under detailed studying, in particular, using model systems, as shown by the fact that the BFB cycle is part of the process of chromothripsis formation [38]. Also in this work, it was suggested that the implementation of chromothripsis by BFB cycles is more typical for oncogenesis, although it is possible in embryo development.

### 4.3. Abortive Apoptosis

The abortive apoptosis (Figure 4) is considered as the third mechanism for the onset of chromothripsis in cells [39,40]. The key regulator of apoptosis is the p53 protein, which is transcribed by the *TP53* gene. It has been found that the apoptosis mechanism is disrupted with the *TP53* mutation. If a cell with *TP53* mutation remains viable with the accumulation of chromosomal mutations, it can lead to chromothripsis. Thus, in lymphocytic leukemia, *TP53* mutations can be combined with chromothripsis [40]. Observation of a higher frequency of chromothripsis in hyperploid medulloblastomas, compared with diploid ones, made it possible to establish a connection between chromothripsis and cell hyperploidization. Moreover, it has been suggested that hyperaneuploidization may be a factor in the development of chromothripsis [41].

Now, three ways of the formation of chromothripsis have been put forward, but it can be assumed that there are other mechanisms of its occurrence, including the implementation of several mechanisms simultaneously.

## 5. Chromothripsis Detection Methods

The identification and description of the chromothripsis characteristics became possible due to modern methods of the chromosomal analysis.

### 5.1. Next-Generation Sequencing (NGS)

**Figure 5:**
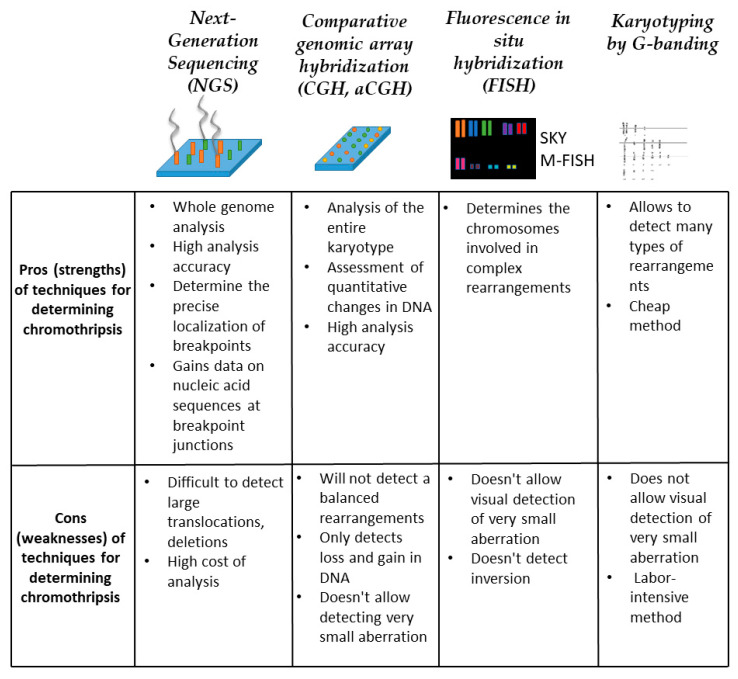
Methods for chromothripsis detection. For each method, conditional strengths and weaknesses are described within the framework of detecting multiple chromosomal rearrangements.

This is one of the most effective methods for detecting structural variations in the genome [42,43]. The method is based on the amplification of a multitude of short sections of genes, in their totality, covering the entire genome, followed by their sequencing. Post-processing of the obtained results using specialized software allows you to compare the analyzed sequins with the reference DNA. Such an analysis makes it possible to identify both numerical and structural alterations in the genome. However, NGS has its limitations, namely, the method cannot identify copy-neutral options and breakpoints for structural alterations. Despite the high cost and methodological difficulties, sequencing is widely used in chromothripsis research (Figure 5).

### 5.2. Comparative Genomic Array Hybridization (CGH, aCGH)

This is another effective technique often referred to as molecular karyotyping or microchip chromosome analysis. Copy number analysis can detect deletions and duplications, and determine their exact location and size in the genome (Figure 5). The resolution of this method is sufficient to detect submicroscopic aberrations. aCGH is based on a direct comparison of two DNA samples to detect changes in the copy number in the genome. During the analysis, test DNA and reference DNA are cut into small fragments, after which the fragments are labeled with a dye, for each sample its own dye. After that, hybridization of the labeled DNA on the chip occurs. Single-stranded DNA probes are applied to the chip in a specific sequence, individual for each chip. After hybridization has taken place on the chip, the fluorescence emitted from each spot is measured. The number of copies of a particular gene is determined by the ratio of the intensity of the emitted signal of two dyes (reference and test). Thus, the aCGH method allows detecting abnormalities in the copy number of genes. To increase the information content of the method, aCGH was combined with the method of single-nucleotide polymorphism on chips (SNP) [44]. On SNP, chip analysis reveals common biallelic polymorphisms in the genome. In this case, the analysis chips are supplemented with SNP-specific probes. Each of the two possible alleles at each SNP locus is amplified using a specific color. In this case, the binding of the labeled products to the probes on the chip also occurs. Then, the chip is read to determine the fluorescence intensity for each SNP. As a result of combining the aCGH and SNP methods, it is possible to determine not only the number of DNA copies but also the heterozygosity of the studied samples. For example, on chromosomes of chromothripsis, the regions with the lowest copy number usually show a loss of heterozygosity. However, the aCGH method has a number of significant limitations. It cannot detect balanced structural chromosomal aberrations or determine the order and orientation of derived chromosomal segments [45,46].

### 5.3. Fluorescence In Situ Hybridization (FISH)

Cytogenetic studies explore a variety of FISH techniques, each of which aims to specifically identify the structure of a derived chromosome. In the case of chromothripsis, spectral karyotyping (SKY) and multicolor FISH analysis (M-FISH) are particularly informative (Figure 5). These methods use probes for whole chromosomes. Thus, using these methods makes it possible to identify all chromosomes involved in complex chromosomal rearrangements. Along with M-FISH and SKY, the method of multicolor FISH banding (MCB-FISH) is used. This method allows one to determine the structure of an aberrant chromosome by segment-specific banding of chromosomes [45]. Often, when analyzing cells with chromothripsis, a combination of SKY and FISH with locus-specific probes is used to determine the structure of the derived chromosome [8]. Also, this combination allows for determining the structure of double minutes, which are often observed in cells with chromothripsis. The undoubted advantage of these methods is that the analysis takes place at the level of individual (single) cells, which allows both to detect rare aberrations in a population and to assess the karyotype of a heterogeneous population.

### 5.4. Karyotyping by G-Banding

To detect chromothripsis in the clinic, the method of karyotyping by G-banding of peripheral lymphocytes is used (Figure 5). With this method, numerical and structural chromosomal abnormalities can be identified. This method is also carried out on individual cells. However, the resolution of the light microscope and high labor intensity are the limitations of this method [46]. In the case of analysis of cells with chromothripsis, the complex nature of multiple changes in chromosomes can also be a limitation of the widespread use G-banded chromosomes for analysis. In this regard, for the identification and structural analysis of chromothripsis rearrangements, it is necessary to use an integrated approach that includes several methods of genetic analysis at once.

## 6. Prevalence of Chromothripsis

Since chromothripsis was initially detected in a patient with lymphocytic leukemia [8], the initial study of chromothripsis focused on cancer patients. At first, it was believed that chromothripsis in cancer cells is a rare phenomenon—3–5%.

To date, it has been shown that the incidence of chromothripsis in cancerous tumors is significantly higher than previously assumed and reaches 100% for some types of cancer [47,48]. So in a recent study, it was found that chromothripsis was detected in 100% cases of malignant tumors of the peripheral nerve sheath, and in 71% cases of germ cell tumors [48]. Chromothripsis is regularly found in blood cancer, cancer of the central nervous system, in soft tissue tumors and carcinomas [49,50], in osteosarcoma and glioblastoma [8,51,52,53,54,55]. It was found that the incidence of chromothripsis in cancer is significantly higher in patients with hereditary genetic disorders associated with gene mutations in the cell cycle and DNA repair [56,57]. Thus, we can put forward the assumption that for those types of cancer in which there is a high frequency of occurrence of chromothripsis, exactly chromothripsis is the driving force behind the formation and development of such tumors.

In addition to cancer cells, chromothripsis has been found in benign tumors. Thus, the phenomenon of chromothripsis has been described in uterine leiomyoma cells [58,59,60], and in meningioma [61,62]. The uterine leiomyoma is a benign tumor of the uterine myometrium, which is characterized by a high frequency of chromosomal abnormalities. Interestingly, chromothripsis with deletions (from 43 to 13,647 kbp) was found in uterine fibroids. However, the cells of this sample of uterine fibroids cultivated in vitro were characterized by a normal karyotype [60].

While studying the genomes of patients with severe congenital anomalies in whom chromothripsis was observed [63], it was revealed that each of the patients inherited chromothripsis from their mother. At the same time, it was surprising that the mothers themselves were healthy, although they had a largely balanced chromothripsis in their genomes. Some of the defective chromosomes from mothers were passed on to their children, resulted in imbalance, which most likely was the cause of their illness.

The phenomenon of chromothripsis was also found in germ cells. It is interesting to note that significant differences were found between embryonic chromothripsis and chromothripsis in cancer [64]. Patients with a congenital disorder often have a more balanced form of chromothripsis [65]. The number of abnormalities in embryonic chromothripsis is usually lower than in cancer [66]. One of the explanations for this is the assumption that most of the deviations are eliminated during fetal development.

Currently, the presence of chromothripsis is not limited to the species Homo sapiens. Such rearrangements have been recorded in the nematodes (*Caenorhabditis elegans*) [67], (*Arabidopsis thaliana*) [68] and grapes (*Vitis vinifera*) [69]. It can be assumed that chromothripsis can be considered as one of the mechanisms of genetic variation.

Thus, we can conclude that the phenomenon of chromothripsis is widespread in nature and even happens in the plant kingdom. At the same time, chromothripsis most often occurs in cancer cells. It can be concluded that this phenomenon has a direct impact on the oncogenic cell transformation.

## 7. Contribution of Chromothripsis to Oncogenesis Process

Since in most cases of the occurrence of chromothripsis researchers dealt with cancer cells, in this review we would like to separately consider the contribution of this phenomenon to the process of oncogenesis. It is obvious that chromothripsis contributes to the development of cancer by disrupting the balance between oncogenes and tumor suppressor genes.

The main question that arose immediately after the discovery of chromothripsis was associated with the effect of chromothripsis on cell proliferation. Usually, somatic cells undergo apoptosis upon significant DNA damage. However, with significant DNA damage, in particular chromothripsis, the activity of tumor suppressor genes can be disrupted and oncogenes can be activated [35,70,71], which promotes the survival of cells with massive chromosome reorganization. Rausch with colleagues found a link between massive genomic rearrangements consistent with chromothripsis and *TP53* mutation. This work was carried out on tumors carrying *TP53* mutations [56]. Further analysis of tumor development once again confirmed a strong relationship between mutations in *TP53* and chromothripsis in cancer: medulloblastoma [72,73], acute myeloid leukemia [56,74,75,76,77], myelodysplastic syndromes [78], glioblastoma [79,80], hepatocellular carcinoma [81] and bladder cancer [82]. Since *TP53* is required for the induction of cell cycle arrest, DNA repair, and apoptosis after DNA damage, inactivation of *TP53* allows the uncontrolled proliferation of cells with DNA damage as a result of chromothripsis. In connection with recent studies, it has been suggested that p53 mutation apparently occurs initially, and then chromothripsis occurs. Perhaps the p53 mutation is the cause, and chromothripsis is the result of this mutation. The interrelation of p53 dysfunction and chromothripsis plays an important role in oncogenesis [83]. At the same time, it was found that just 40% of tumors had a p53 mutation, and chromothripsis can also be observed in cells with normally functioning p53 [84]. In a recent study, it was found that chromothripsis can cause the loss of repair genes in cancerous tumors [83]. Another feature of cancers with chromothripsis—in such tumors is often observed the amplification of oncogenes localized in double minutes [8,48,56,85]. This can make it difficult to treat these tumors. A number of studies have found a significant increase in the number of chromothriptic events in hyperploid cells compared to diploid cells in vitro [41,86,87]. It is interesting to note that among the many cases of chromothripsis, patterns of involvement of certain chromosomes or their regions in complex rearrangements are revealed. In a recent study by Dr. Voronina and colleague, it was found that in most cases of chromothripsis, the telomeric regions of chromosomes were not affected by chromothripto-like rearrangements, when in almost half of the cases studied, the centromeric region was involved in such rearrangements [48]. When screening 22,347 cancer genomes, chromothripto-like patterns were more often detected on chromosomes 8, 11, 12, and 17 [75]. In acute myeloid leukemia, chromothripsis is observed in almost all chromosomes, most often in chromosome 7 [76]. Among the cases of chromothripsis found in pancreatic cancer, 11% occurred on chromosome 18 and 8% on chromosome 12. The chromothripsis mainly affected chromosomes 5, 12, and 17 in osteosarcomas [53]. It was found that chromosome 17 was involved in chromothripsis in all subtypes of breast cancer [88,89], in glioblastomas were involved chromosomes 9 and 12 [90]. More than half (54%) of the chromothripsis identified in bladder cancer was associated with chromosomes 4, 5, and 6 [82]. Chromothripsis associated with changes in chromosome 13 is a recurrent disorder of high-risk myelodysplastic syndromes [78]. On the one hand, the predominant involvement of certain chromosomes in chromothripsis may be due to a set of genes localized in these chromosomes. On the other hand, the increased prevalence of chromothripsis in certain chromosomal regions may be due to the fact that these regions are structurally more fragile. Recently, an interesting suggestion has been made that perhaps the preferential involvement of certain chromosomes in different cancer types is due to the selective advantage of such cells, rather than more frequency of chromothripsis on certain chromosomes [48].

A number of researchers have attempted to consider the phenomenon of chromothripsis as a predictive marker for the development and therapy of cancer. It was suggested it to be a positive predictive marker of chemotherapy for metastatic collateral cancer [91]. The authors hypothesized that cancer cells exhibiting radical DNA rearrangements such as chromothripsis are more susceptible to nucleic acid-damaging therapy with 5-FU and oxaliplatin. Realigned areas can potentially indicate the vulnerability of chromothripsis tumors, thereby identifying targets for treatment.

At the same time, a number of researchers have shown that chromothripsis is more common in cases of aggressive cancer and is associated with poor patient survival. The chromothripsis is a poor prognostic marker in such types of cancer as medulloblastoma [73], acute myeloid leukemia [77], neuroblastoma [92]. In the study by Dr. Shoshani and colleagues, it was found that having double minutes in cancer cells with chromothripsis is a poor predictor for patients [93]. In such cases, resistance to cancer therapy is often observed. It can be explained by the fact that either multidrug resistance develops very quickly in such tumors, or anticancer therapy does not work due to the localization of a large number of oncogenes in double minutes [83].

Despite the information available to date about the prevalence of chromothripsis in nature and its relationship with tumor cells, at the moment we are still at the stage of collecting information. To use this type of aberration as a prognostic factor in anticancer therapy, additional studies are definitely needed with the attraction of a large amount of data. Nevertheless, the fact of the presence in nature of genome reconstruction by chromothripsis, as well as an increase in the frequency of occurrence of chromothripsis in cancer cells, make one think about the importance of this phenomenon and the need to massively research in this direction.

## 8. Conclusions

The discovery of chromothripsis happened relatively recently, but it turned the idea of genetic variability upside down and made great adjustments to the mechanisms of oncogenesis and hereditary diseases. The originality of this phenomenon attracts more and more attention from scientists and physicians. In our understanding, the discovery of chromothripsis led to the discovery of an alternative pathway of oncogenesis that does not destroy the general theory of oncogenesis but complements it. With further research in this area, we await the elucidation of additional mechanistic pathways and cellular processes underlying chromothripsis. Understanding these processes may lead to strategies for developing new preventive and therapeutic measures to combat cancer.
